# A novel heterozygous mutation c.680A>G (p. N227S) in *SLC34A1* gene leading to autosomal dominant hypophosphatemia: A case report

**DOI:** 10.1097/MD.0000000000015617

**Published:** 2019-05-17

**Authors:** Xiang Chen, Ying Xie, Shan Wan, Jin Xu, Bei Cai, Yi Zhang, Xijie Yu

**Affiliations:** a Laboratory of Endocrinology and Metabolism, Department of Endocrinology and Metabolism, National Clinical Research Center for Geriatrics, West China Hospital, Sichuan University; b Department of Laboratory Medicine, West China Hospital, Sichuan University; c Core Facility of West China Hospital, Sichuan University, Chengdu, China.

**Keywords:** autosomal dominant hypophosphatemia, heterozygous mutation, hypophosphatemia, SLC34A1

## Abstract

**Rationale:**

Currently, the relationship between heterozygous mutations in SLC34A1 and hypophosphatemia is controversial. Here we report an autosomal dominant hypophosphatemia pedigree carrying a novel heterozygous mutation in SLC34A1.

**Patient concerns:**

The proband is a 32-year old young man, presented with progressive pain and weakness in his lower extremities for more than 5 years. The proband showed persistent hypophosphatemia and low TmPO4/GFR values, indicating renal phosphate leak. His grandfather, father, and one of his uncles showed the similar symptoms.

**Diagnoses:**

Autosomal dominant hypophosphatemia.

**Interventions and outcomes:**

Phosphorus supplement was prescribed to the proband and his affected uncle. Both their serum phosphorus levels recovered to normal and their symptoms such as back pain and lower extremity weakness were completely relieved. Whole exome sequencing was performed to identify disease-causing mutations in proband.

**Lessons:**

A novel heterozygous missense mutation c.680A>G (p. N227S) in exon 7 of SLC34A1 was found in proband by whole exome sequencing, which was also found in other 4 family members of this pedigree. Our report of an autosomal dominant hypophosphatemia pedigree with 5 mutant carriers enriches the clinical phenotype caused by the SLC34A1 mutations and further affirms the heterozygous mutations are causative for hypophosphatemia.

## 1 Introduction

NaPi-IIa, encoded by SLC34A1, is a key phosphate transporter in murine animal models. It is still uncertain whether it plays a key role in humans. It has been definitively established that homozygous mutations of SLC34A1 are associated with autosomal recessive Fanconi syndrome and idiopathic infantile hypercalcemia and nephrocalcinosis.^[1,2]^ However, it is still contradictory whether the heterozygous SLC34A1 mutations lead to renal phosphate leak and hypophosphatemia.^[3]^ Prie‘ and colleagues have reported that 2 heterozygous mutations (A48F and V147M) in SLC34A1 may be responsible for hypophosphatemia and urinary phosphate loss in persons with urolithiasis or bone demineralization,^[4]^ which, however, was refuted by another study conducted by Virkki et al In their study, Virkki et al found no dominant negative effect of these variants and suggested these variants may be polymorphisms.^[5]^ In another study, Lapointe et al also indicates that genetic variants of NaPi-IIa are not associated with phosphate leak and hypercalciuria in a large cohort of hypercalciuric stone-forming pedigrees.^[6]^ Here we report an autosomal dominant hypophosphatemia pedigree carrying a novel heterozygous mutation in SLC34A1, which segregates with the disease phenotype.

## 2 Case report

**Figure 1:**
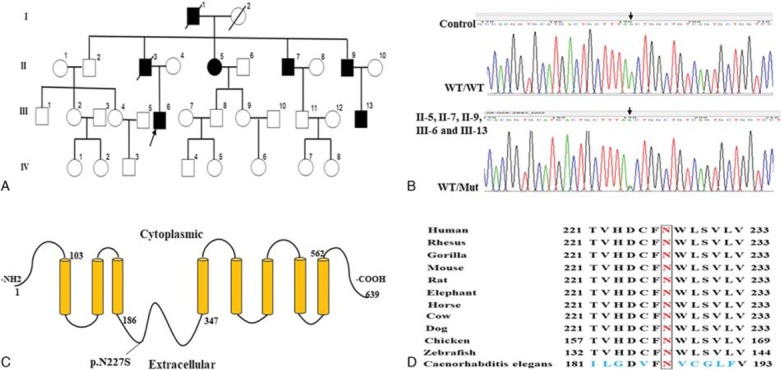
Pedigree of a hypophosphatemia family and genetic analysis. (A) Family tree of an autosomal dominant hyponatremia pedigree. Arrow denotes the proband (III-6), solid symbols represent the affected individuals (I-1, II-3, II-5, II-7, II-9, III-6, and III-13). Symbols with diagonal slashes denote deceased individuals. (B). A missense mutation c.680 A > G (p. N227S) in SLC34A1 was found in proband (III-6), II-5, II-7, II-9, and III-13. WT, wild type; Mut, mutation. (C). Model of heterozygous c.680 A > G (p. N227S) in SLC34A1. (D). Conservation of the asparagine residue among vertebrates and invertebrates.

The study was approved by the ethics committee of West China Hospital of Sichuan University. Informed written consent was obtained from the patients for publication of this case report and accompanying images. The proband (III-6) is a 32-year old young man. He suffered from progressive pain and weakness in his lower extremities for more than 5 years. The patient gradually became unable to walk about 1 year before. His grandfather, father, and one of his uncles also showed the similar symptoms. His grandfather (I-1) and father (II-3) presented with the similar symptoms around the age of 40, gradually unable to walk, and eventually died with unknown causes. His uncle (II-9, 47 years) suffered from low back pain, lower extremity weakness and inability to run for about 1 year. The patient's height was 153 cm, and body weight was 61 kg. Physical examination found external rotation of his hips and internal rotation of his knees. Biochemical examination revealed significantly lower serum phosphate levels and higher bone-specific alkaline phosphatase (B-ALP) and parathyroid hormone (PTH) levels (Table 1). Serum levels of type I collagen C-telopeptide (CTx), 25-OH-VD, calcium and magnesium were in the normal range (Table 1). His urinary phosphorous, calcium, and magnesium levels were also decreased (Table 1). The mean bone mineral density, T scores, and Z scores of the lumbar L1-L4 were 0.654 (g/cm^2^), −3.6, and −3.4, respectively. The mean bone mineral density, T scores and Z scores of the femoral neck were 0.835 (g/cm^2^), −1.1, and −1.0, respectively. X-ray showed osteoporosis in vertebral and pelvic, suspected old fracture changes in bilateral pubic ramus, right rami superior ossis pubis, and left femoral neck. His uncle (II-9) also presented with hypophosphatemia and bilateral femoral neck fractures (Table 1, Fig. 1).

**Table 1:**
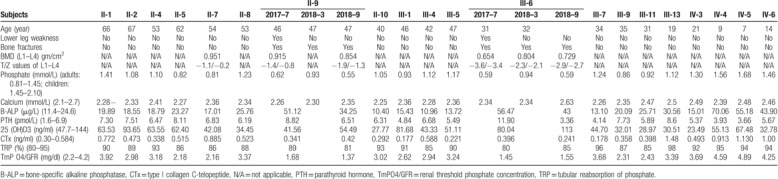
Clinical and biochemical results.

Serum, plasma, and urine specimens were collected in the morning after overnight fasting from proband and his relatives. The phosphate fractional tubular reabsorption (TRP) and the tubular maximum reabsorption threshold of phosphate per glomerular filtration rate (TmPO4/GFR) were calculated as previously reported.^[7]^

## 3 Genetic analysis

Whole exome sequencing (WES) of the proband was performed and the detected possible mutations were further analyzed in DNA samples from his relatives (Joy Orient Translational Medicine Research Center Co., Ltd. Beijing, China).

## 4 Results

### 4.1 Clinical and laboratory findings

The proband and one of his uncles (II-9) showed persistent hypophosphatemia. The serum phosphate levels in another uncle (II-7, 54 years) and aunt (II-5, 62 years) were in the lower limit of the normal range (Table 1). The PTH levels in the proband, II-5, and II-9 were increased, and the PTH levels in II-7 were in the higher limit of the normal range. The TRP values in proband were in the lower limit of the normal range. The TmPO4/GFR values in proband, II-5, II-7, and II-9 were low. Our data suggested that TmPO4/GFR may be more sensitive than TRP. Both II-5 and II-7 did not show the similar symptoms as proband.

The proband and II-9 were prescribed phosphorus supplement. Both their serum phosphorus levels recovered to normal and their symptoms such as back pain and lower extremity weakness were completely relieved. The DEXA scan performed 8 months later showed obvious improve in bone mass of the proband. His bone mineral density, T and Z scores of lumbar vertebrae, L1 to L4 were 0.804 (gm/cm^2^), −2.3, and −2.1, respectively. The mean bone mineral density, T scores, and Z scores of the femoral neck were 1.134 (g/cm^2^), 1.2, and 1.4, respectively (Table 1).

### 4.2 Mutational analysis

A novel heterozygous missense mutation c.680A>G (p. N227S) was found in exon 7 of SLC34A1 (NM_003052) (Fig. 1B). The A→G substitution was further confirmed by Sanger sequencing (Fig. 1B). Other hypophosphatemia-related genes, including *FGF23*, *DMP1*, *PHEX*, *CYP27B1*, *CYP2R1*, *VDR*, *SLC34A3*, *CLCN5*, *ENPP1*, and *OCRL* were normal. This mutation was not found in OMIM, HGMD, and Clinvar database, and was also not included in population databases, including 1000 Genomes, dbSNP, Exome Variant Server, and ExAC Browser. This mutation was predicted to be deleterious by Provean, Polyphen2, Sift, and Mutation taster. II-5, II-7, II-9, and III-13 (19 years) also carried this mutation.

## 5 Discussion

This is an autosomal dominant hypophosphatemia pedigree. A heterozygous missense mutation c.680A>G (p. N227S) in SLC34A1 was found in this pedigree. The following findings support the causative role of the SLC34A1 N227S mutation in the pathogenesis of the disease in our pedigree: this is a novel mutation and is absent in multiple public databases; this mutation segregates with the disease phenotype and is predicted to be deleterious by different in silico prediction programs; the asparagine residue is highly conserved across species; other known hypophosphatemia-related genes were excluded by WES.

*SLC34A1* gene mutations lead to various overlapping clinical syndromes. Homozygous SLC34A1 mutations cause autosomal recessive Fanconi syndrome, nephrocalcinosis and kidney insufficiency, and infantile idiopathic hypercalcemia with vitamin D hypersensitivity.^[2,8,9]^ Both recessive and dominant inheritance of *SLC34A1* gene mutations have been found in families with nephrolithiasis/nephrocalcinosis.^[10]^ Heterozygous mutations of SLC34A1 have been reported to be linked with hypophosphatemia, although with inconsistent results.^[4,5]^ It is not uncommon that different genetic patterns caused by the same gene.

This pedigree is characterized by diverse and variable clinical manifestations. Symptoms usually began to occur in middle age (about 30–40 years). Severe conditions include weakness of the lower extremities, fractures, inability to walk, and even death, if without prompt treatment. Some carriers (II-5 and II-7) only showed slightly decreased TmPO4/GFR values with normal but in the lower limit of reference ranges of serum phosphorus levels, without the above mentioned clinical manifestations. III-13 (19 years) is totally normal, possibly due to his young age or incomplete penetrance in dominant autosomal inheritance. The diverse phenotypes may be due to age, environmental factors, or other genetic differences. To the best of our knowledge, this is the largest pedigree that has been reported to be associated with the SLC34A1mutations, with 5 carriers of mutation, which further supports the causative role of the SLC34A1 mutation in hypophosphatemia.

In conclusion, we report a novel heterozygous mutation c.680A>G (p. N227S) in SLC34A1 in an autosomal dominant hypophosphatemia pedigree, which enriches the clinical phenotype caused by the mutations of SLC34A1 and affirms the heterozygous mutations are causative for hypophosphatemia.

## Author contributions

**Formal analysis:** Xiang Chen.

**Funding acquisition:** Xijie Yu.

**Investigation:** Xiang Chen, Ying Xie, Shan Wan.

**Methodology:** Xiang Chen, Ying Xie, Shan Wan, Jin Xu, Bei Cai, Yi Zhang.

**Project administration:** Xiang Chen, Xijie Yu.

**Writing – original draft:** Xiang Chen.

**Writing – review & editing:** Xijie Yu.

Xiang Chen orcid: 0000-0002-8089-3667.
